# The immunological function of extracellular vesicles in hepatitis B virus-infected hepatocytes

**DOI:** 10.1371/journal.pone.0205886

**Published:** 2018-12-31

**Authors:** Masatoshi Kakizaki, Yuichiro Yamamoto, Suemi Yabuta, Natsumi Kurosaki, Tatehiro Kagawa, Ai Kotani

**Affiliations:** 1 Division of Hematological Malignancy, Institute of Medical Sciences, Tokai University, Isehara, Japan; 2 Division of Gastroenterology and Hepatology, Department of Internal Medicine, Tokai University School of Medicine, Isehara, Japan; 3 Department of Hematology and Oncology, Tokai University School of Medicine, Isehara, Japan; The University of Tokyo, JAPAN

## Abstract

Hepatitis B virus (HBV) generates large amounts of complete and incomplete viral particles. Except for the virion, which acts as infectious particles, the function of those particles remains elusive. Extracellular vesicles (EVs) have been revealed to have biological functions. The EVs which size are less than 100 nm in diameter, were collected from HBV infected-patients. These vesicles contain, complete and incomplete virions, and exosomes, which have been recently shown to be critical as intercellular communicators. Here, the effects of the exosome, the complete, and the incomplete particles on the target cells were investigated. These particles are endocytosed by monocyte/macrophages and function primarily to upregulate PD-L1. The functions and composition of the EVs were affected by nucleotide reverse transcriptase inhibitors (NRTIs), suggesting that the EVs are involved in the pathogenesis of HBV hepatitis and clinical course of those patients treated by NRTIs.

## Introduction

The pathogenesis and clinical manifestations of the hepatitis B virus (HBV) occur due to the interaction of the virus with the host immune system, which leads to liver injury and potentially cirrhosis and hepatocellular carcinoma [1–3].

A major characteristic of HBV is the secretion of large amounts of complete and incomplete viral particles. The extracellular vesicles (EVs), which have been revealed to have biological functions, are less than 100 nm in diameter and are collected from HBV-infected patients. These vesicles contain exosomes, which have been recently revealed to play a critical role in intercellular communication and the viral particles. The incomplete form mainly consists of two types. The first type is the classical HBV surface antigen (HBsAg) spheres and filaments that are 20 nm in diameter (HBsAg particles). These subviral particles are composed of only the viral surface proteins and are found in the blood of infected individuals at up to 100,000-fold in excess over the complete virions (at 10^14^/mL) [4]. The second class is the recently discovered empty genome-free virions, which contain the surface proteins enclosing the viral capsid but no genome, and are typically found at 100-fold higher levels over the complete virions in the blood of infected individuals [5, 6]. In addition, putative particles containing HBV RNA at much lower levels than the other particles (100- to 1000-fold lower than the complete virions) have also been recently reported [7–10]. In contrast, the complete HBV virions, which are spheres 42 nm in diameter, are routinely found in the blood of infected patients at a concentration of 10^9^ virions/mL. The assembly begins with the packaging of the viral pregenomic RNA (pgRNA), as a complex with the viral reverse transcriptase (RT) protein, into an icosahedral capsid, which is 30 nm in diameter and is composed of the viral capsid or core protein (HBc; also called HBV core antigen or HBcAg). Within the capsid, RT first converts pgRNA to a single-stranded (SS) (minus-sense) DNA, and subsequently to a partially double-stranded (DS), relaxed circular DNA (RC DNA) [11]. Nucleocapsids (NCs) with pgRNA and SS DNA are considered immature as they are incompetent for the envelopment and secretion of virions. In contrast, RC DNA-containing NCs are considered mature and are selected for envelopment by the viral envelope or surface proteins (HBsAg) and are secreted extracellularly as complete virions [12–15]. As a result, a complete HBV virion contains an outer envelope enclosing an inner capsid, which in turn encloses the RC DNA genome. Except for the complete virion, which infects hepatocytes, these particles, though they are abundantly secreted, have not been fully investigated for their role in viral–host interaction.

The nucleotide reverse transcriptase inhibitors (NRTIs), which are currently the most commonly used drugs for the treatment of HBV hepatitis as well as HIV infections, prevent HBV replication by blocking HBV reverse transcriptase by inhibiting HBV DNA production from HBV pregenomic RNA. Clinically, inhibition of HBV replication by NRTIs efficiently leads to a decrease in HBV DNA levels in the blood and chronic liver inflammation [16]. However, when the administration of NRTIs is discontinued, a relapse of the HBV DNA levels and liver inflammation frequently occurs in patients with HBV, and the disease worsens and sometimes develops into fulminant inflammation. Therefore, most patients must continue to take the drug for the rest of their lives. Accordingly, it is postulated that NCs, which contain only pgRNA, accumulate in the cells, because NCs with pgRNA and SS DNA are considered immature as they are incompetent for envelopment and secretion as virions.

EVs, which contain virus-coding RNA and proteins, are secreted from the infected cells and transferred to the target cells. Epstein Bar virus EBV encodes its own small RNAs packed into the EVs, which are then used to manipulate the immune system establish a tumour microenvironment [17]. HBV has been reported to secrete exosomes, which contains HBV RNA and proteins [18]. Furthermore, HBV DNA has been reported to be encapsulated by the exosome [19]. Accordingly, the function and biological significance of the majority of the EVs except for the virion and exosome have been elusive.

Here, we investigated the target cells and function of the EVs secreted from HBV-infected hepatocytes. Furthermore, the influence of NRITs, which inhibits RT to make the complete virion, on the composition of the EVs. We revealed that EVs target peripheral blood monocytes and significantly upregulated programmed death ligand-1 (PD-L1), which is a critical immune molecule that downregulates CD69. When the complete virion is depleted by NRTIs, marked changes in the composition and character of the particles were observed. These results suggest that the EVs secreted from HBV infected hepatocytes have critical roles in the host immune systems, and NRITs not only inhibited the generation of the complete virion, but also affected EV biogenesis, which leads to alterations of the host immune environment.

## Materials & methods

### Cells

HepAD38 cells, which express HBV pgRNA under the control of an inducible tetracycline promoter [20], were used in this study. The cells were cultured in DMEM/F12 (Life Technologies, Carlsbad, CA) supplemented with 10% doxycycline-free fetal bovine serum (FBS), 100 U/mL penicillin, and 100 μg/mL streptomycin (Life Technologies) at 37°C humidified air that contained 5% CO2. Furthermore, the repression or maintenance of HBV replication was maintained with or without 1 μg/mL of doxycycline, respectively. We used healthy human blood samples which was approved to IRB reviewer by Tokai University. The consent was informed by written form. PBMCs were isolated from heparinised whole blood of healthy individuals using lymphoprep (Axis-Shield, Oslo, Norway). Isolated PBMCs (2.0 × 10^5^) were cultured in RPMI 1640 medium (Life Technologies) supplemented with 10% (vol/vol) FBS, 100 U/mL penicillin, and 100 mg/mL streptomycin in 24-well plates.

### NRTIs treatment

The NRTIs entecavir (ETV) (Tokyo chemical industry, Tokyo, Japan), lamivudine (LMV) (Wako, Osaka, Japan), and tenofovir (TDF) (Wako) were used. HepAD38 cells with HBV replication (5.0 × 10^6^) were seeded onto a 10-cm dish and exposed to 10 μM ETV, 8 μM LMV, and 65 μM TDF for a total of 9 days, reseeding every 3 days to the original cell density. At 9 days after the NRTIs treatments, culture supernatants were used for EV isolation, and cells were used for western blotting and quantitative PCR analysis.

### EV isolation

HepAD38 cells with or without HBV replication treated with NRTIs were cultured in DMEM/F12 medium for 3 days. Culture supernatants were collected and centrifuged at 1500 ×g for 15 min at room temperature. To thoroughly remove cellular debris, the supernatants were filtered through a 0.22 μm filter (Merck Millipore, KGaA, Darmstadt, Germany). For EV preparation, the filtered supernatants were ultracentrifuged at 110,000 ×g for 70 min at 4°C. The pellets were washed with 8 mL of PBS after ultracentrifugation and resuspended in PBS. Collected EVs were labelled with PKH26 dye (SIGMA-ALDRICH, Saint Louis, Mo). Then, the amount of in EVs were measured by nanophotometer (Implen, Munich, Germany).

### Density gradient separation

To divide the EVs into exosomes, subviral particles, and virions, density gradient centrifugation was performed after ultracentrifugation. Then, 10%, 20%, and 30% iodixanol solutions were prepared by mixing Optiprep (Axis-Shie) with buffer containing 0.25 M sucrose, 10 mM Tris at pH 8.0, and 1 mM EDTA, with a final pH set of 7.4. EV pellets were resuspended in 2.4 mL of 30% iodixanol solution, and resuspended EVs were transferred to a NVT90 rotor tube (Beckman). Subsequently, 1.3 mL of 20%, and 1.2 mL of 10% iodixanol solutions were successively layered on top of the suspension, and tubes were centrifuged for 75 minutes at 4°C at 97,500 ×g (35,000 rpm)

### Flow cytometry

EVs, exosomes, subviral particles, or virions were added to PBMC cultures. After 24 hours, the cells were collected. Subsequently, the cells were washed twice in PBS and resuspended in FACS buffer, which contained 2% FBS in PBS. To assess the expression levels of PD-L1, PD-L2 and CD69, the cells were incubated with APC anti-human PD-L1 (Biolegend, San Diego, CA), PE/Cy7 anti-human PD-L2 (Biolegend), and PE/Cy7 anti-human CD69 (Biolegend) for 20 min at room temperature, followed by 3 washes with FACS buffer. Subsequently, flow cytometry was performed using FACS Verse (BD Bioscience, San Jose, CA). Date were analysed using the FlowJo software (Tree Star, Jackson, OR).

### Western blot analysis

Cells (1.0 × 10^6^) and EV pellets were lysed in radioimmunoprecipitation assay buffer (Wako) for 30 min on ice, and cell lysates were centrifuged to remove debris. Total protein from EVs lysate were measured using DC protein assay kit (Bio-Rad Laboratories, Hercules, CA). Protein samples were electrophoresed on a 12% SDS–polyacrylamide gel and blotted onto PVDF membranes (Bio-Rad Laboratories). The blots were blocked with PVDF Blocking Reagent (TOYOBO, Osaka, Japan) for 1 h at room temperature. The blocked membranes were incubated with the appropriate primary antibodies diluted in Can Get Signal solution 1 (TOYOBO) for 1 hour at room temperature, followed by incubation with horseradish peroxidase (HRP)-conjugated corresponding secondary antibodies diluted in Can Get Signal solution 2 (TOYOBO) for 1 hour at room temperature. Subsequently, the blots were incubated with Immobilon Western Chemiluminescent HRP Substrate (Merck Millipore) for a few seconds, and chemiluminescence was detected using the ChemiDoc Touch system (Bio-rad). Densitometric analysis was performed using the Image Lab software (Bio-rad). The following antibodies were used: rabbit anti-CD9 (System Biosciences, Palo Alto, CA), mouse anti-CD63 (Santa Cruz Biotechnology, Santa Cruz, CA), mouse anti-HBV PreS2 (Abcam, Cambridge, UK), mouse anti-HBcAg (Millipore, Temecula, CA), mouse anti-α-tubulin (Sigma), goat anti-rabbit IgG HRP (System Bioscience), and sheep anti-mouse IgG HRP (Sigma).

### Quantitative PCR

Total RNA of HepAD38 cells were isolated using Sepasol-RNA I Super G (Nacalai, Kyoto, Japan). HBV RNA of HepAD38 cells culture supernatant was isolated using QIAamp MinElute Virus Spin kit (QIAGEN, Hilden, Germany) according to the manufacturer’s instructions and treated with DNase 1 (Promega, Madison, WI). Reverse transcription-PCR was performed with the High-Capacity Reverse Transcription Kit (Applied Biosystems, Carlsbad, CA), and qPCR was performed with the THUNDERBIRD SYBR qPCR Mix (TOYOBO) following the manufacturer’s protocol, respectively. The primer used to detect pgRNA was as follows: forward, 5′- GAGTGTGGATTCGCACTCC-3′; and reverse 5′- GAGGCGAGGGAGTTCTTCT-3′.

## Results

### EVs secreted from HepAD38 cells were endocytosed by monocytes and upregulated PD-L1 expression levels

In patients with chronic HBV infection, PD-L1 has been shown to be elevated in circulating monocytes, and therefore, may contribute to ongoing T cell exhaustion [21]. However, the mechanism of PD-L1 elevation in circulating monocytes has not been fully elucidated. Accumulating evidence has shown that EVs secreted from virus-infected cells were endocytosed by dendritic cells or macrophages to induce immune responses in the cells [17, 22, 23]. Therefore, it could be assumed that EVs secreted from HBV infected cells induce PD-L1 expression in the circulating monocytes.

**Fig 1:**
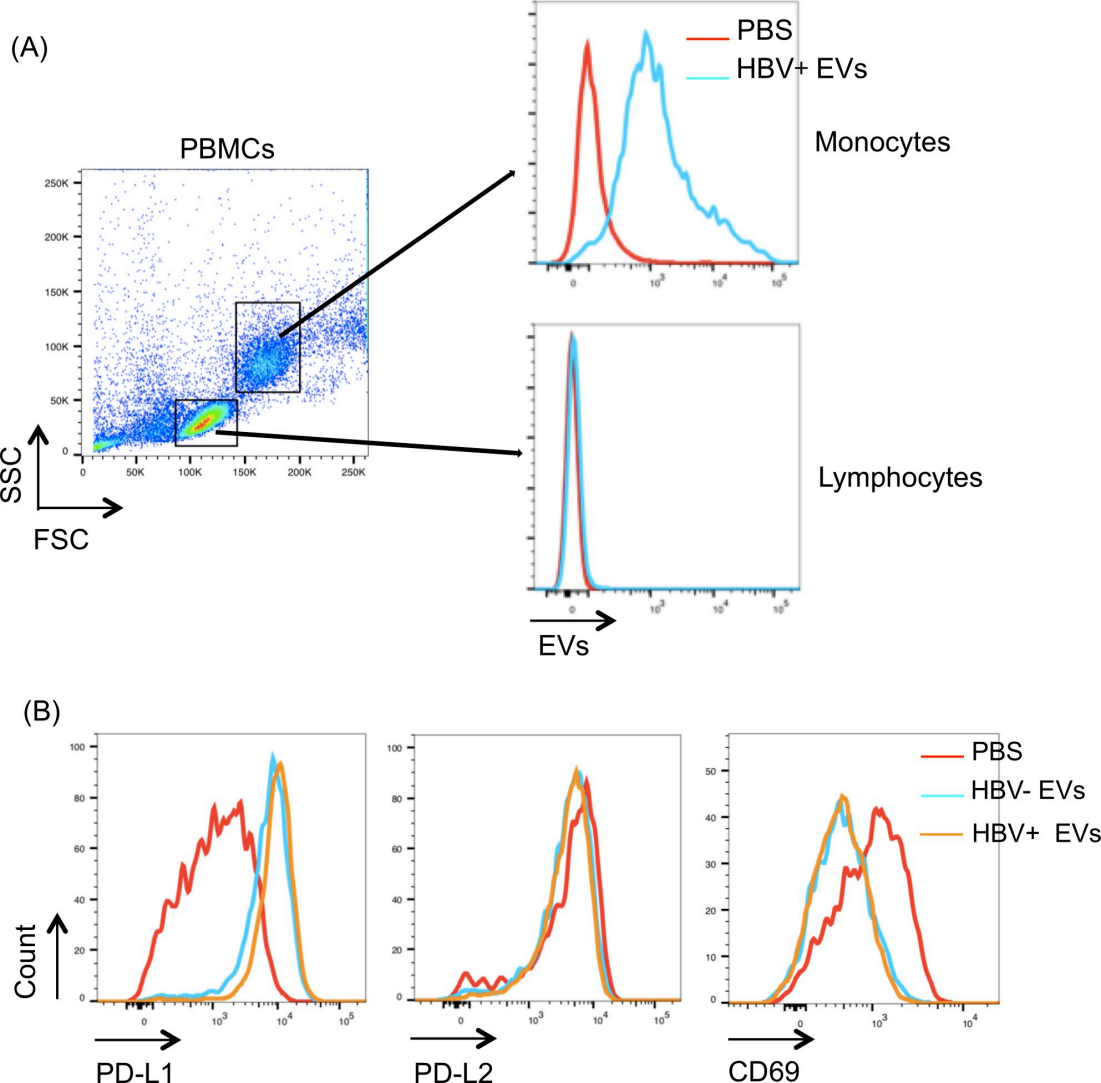
Induction of immune suppression in monocytes by EVs. (A) Extracellular vesicles (EVs) were collected by ultracentrifugation from 10 mL of culture supernatant of HepAD38 cells with or without hepatitis B virus (HBV) replication. The collected EVs were then labelled with PKH26 dye. Peripheral blood mononuclear cells (PBMCs) were then treated with 10 μg of PKH-labelled EVs for 24 hours. Uptake of EVs was evaluated by flow cytometry. (B) PBMCs treated with EVs were stained with PD-L1, PD-L2, and CD69 and analysed by flow cytometry.

First, to investigate whether the monocytes could be regulated by EVs secreted from HepAD38 cells with HBV replication, human PBMCs were treated with EVs in vitro. EVs were isolated from culture supernatant of HepAD38 cells with or without HBV replication by ultracentrifugation. PBMCs were treated with the 10 μg of PKH26-labelled HepAD38 EVs for 24 hours. Flow cytometric analysis showed that lymphocytes did not endocytose the EVs, whereas monocytes selectively consumed the EVs (Fig 1A). Furthermore, regardless of HBV replication, PD-L1 expression was increased, and expression of CD69, which is a marker of activated immune cells, was decreased in monocytes that consumed the EVs (Fig 1B). In particular, EVs secreted from HepAD38 cells with HBV replication upregulated PD-L1 in monocytes (Fig 1B). These results indicated that EVs secreted from HepAD38 cells had the ability to induce immune suppression in monocytes.

### Exosomes, subviral particles, and virions induced immune suppression in monocytes

**Fig 2:**
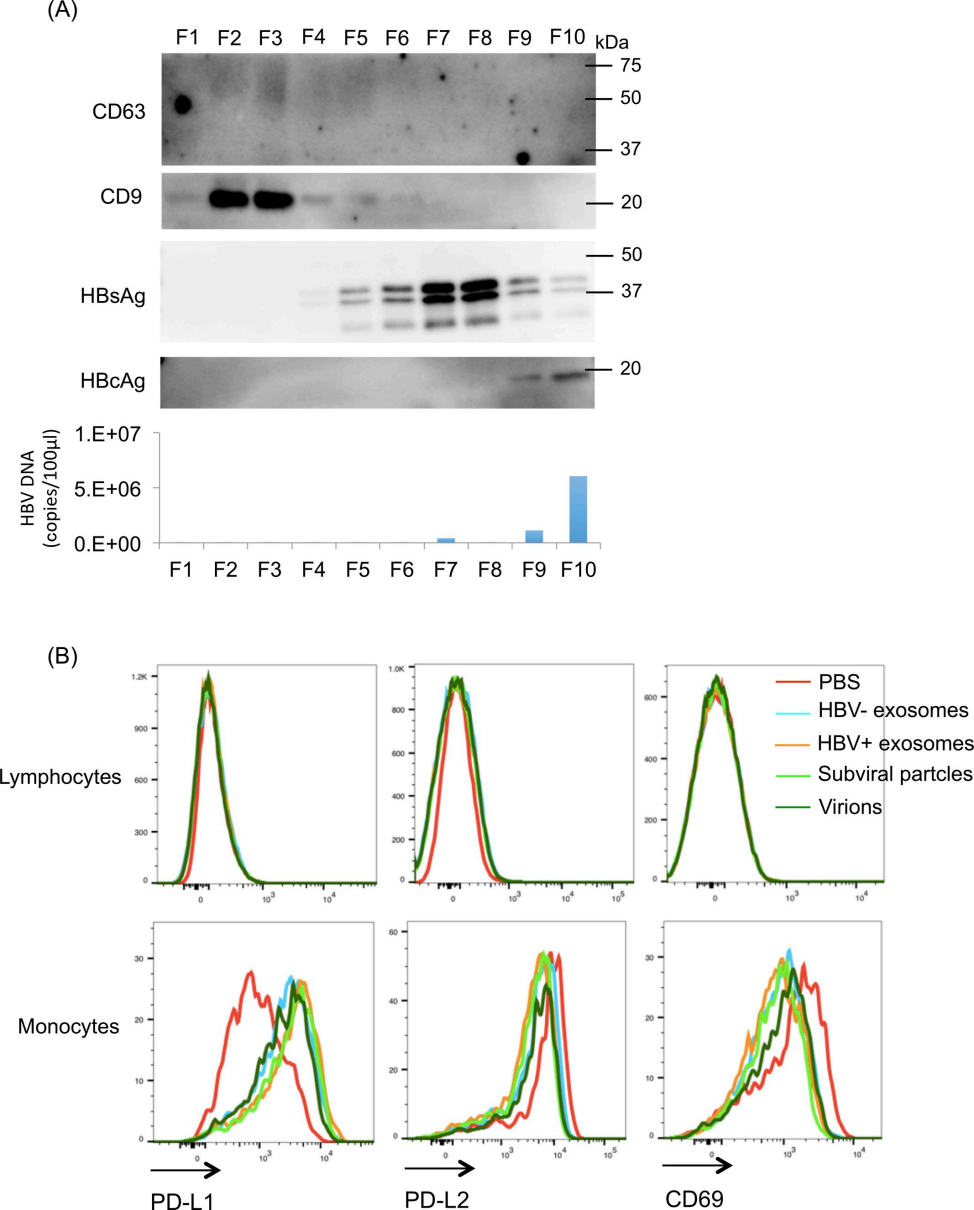
Separation and immunosuppressive effects of exosomes, subviral particles, and virions. (A) Extracellular vesicles (EVs) were collected by ultracentrifugation from 10 mL of culture supernatant of HepAD38 cells with or without hepatitis B virus (HBV) replication. Collected EVs were fractionated by a density gradient separation using iodixanol. Ten fractions were collected, and the presence of CD9, CD63, HBsAg, HBcAg, and HBV DNA in each fraction were analysed by western blotting and quantitative RT-PCR (representative of three experiments). (B) Peripheral blood mononuclear cells (PBMCs) were treated with exosomes, subviral particles, and virions for 24 hours. PBMCs treated with each particle were stained with PD-L1, PD-L2, and CD69 for analysis by flow cytometry.

EVs secreted from HepAD38 cells with HBV replication contain exosomes, subviral particles, and virions. Therefore, we separated EVs into exosomes, subviral particles, and virion using a density gradient, and then treated PBMCs with each particle. Ten fractions were recovered and analysed for the presence of exosome markers, CD9 and CD63, viral proteins, HBsAg and HBcAg, and viral DNA. As shown in Fig 2A, exosomes were found in fractions 2 and 3 (F2 and F3, respectively), subviral particles were found in F4F8, and virions were found in F9 and F10. This result indicated that exosomes, subviral particles, and virions were successfully separated from EVs obtained from HepAD38 cells.

PBMCs were treated with 20 μg of exosomes, subviral particles, or virions for 24 hours. Flow cytometric analysis showed that the expressions of PD-L1, PD-L2, and CD69 did not changed in lymphocytes after treatment with each particle (Fig 2B). However, in monocytes, PD-L1 expression was upregulated, and CD69 expression was decreased after treatment with each particle (Fig 2B). In particular, exosomes and subviral particles secreted from HepAD38 cells with HBV replication strongly upregulated PD-L1 expression and downregulated CD69 expression (Fig 2B). These results indicated that exosomes and subviral particles secreted from HepAD38 cells with HBV replication had a strong immmuno-suppressive effect compared to exosomes secreted from HepAD38 cells without HBV replication.

### EVs secreted from HepAD38 cells treated with TDF strongly induced PD-L1 expression

**Fig 3:**
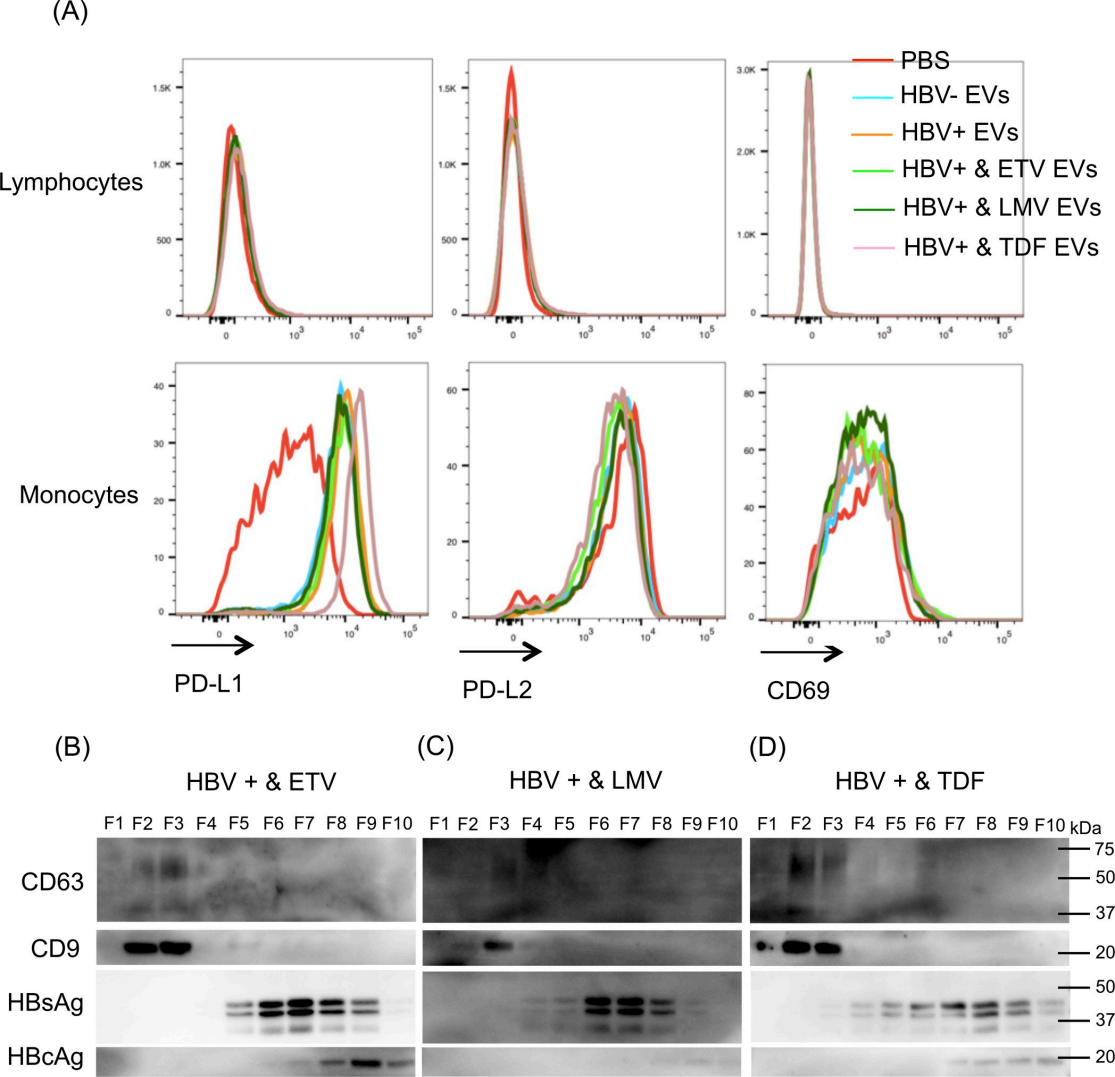
The effects of NRTIs on the extracellular vesicles (EVs) secreted from HepAD38 cells with hepatitis B virus (HBV) replication. (A) HepAD38 cells with HBV replication were treated with entecarvir (ETV), lamivudine (LMV), and tenofovir (TDF) for 9 days. Then, EVs were collected by ultracentrifugation from 10 mL of culture supernatant of HepAD38 cells treated with each NRTIs. PBMCs were treated with each EV for 24 hours and were stained with PD-L1, PD-L2, and CD69 for analysis by flow cytometry. (B–D) EVs collected from HepAD38 cells with HBV replication treated with NRTIs s were fractionated by density gradient separation using iodixanol. Ten fractions were collected, and the presence of CD9, CD63, HBsAg and HBcAg were analysed by western blotting (representative of three experiments).

Next, we investigated whether NRTIs treatments would influence the function of EVs secreted from HepAD38 cells with HBV replication. HepAD38 cells with HBV replication were treated with entecarvir (ETV), lamivudine (LMV), and tenofovir (TDF) for 9 days. Then, EVs were isolated from each culture supernatant by ultracentrifugation, and then PBMCs were treated with 10 μg of each EVs for 24 hours. As shown in Fig 3A, the expressions of PD-L1, PD-L2, and CD69 did not change after treatment with each EV in lymphocytes. However, in monocytes, PD-L1 expression was strongly upregulated after TDF treatment with EVs isolated from HepAD38 cells with intact HBV replication (Fig 3A). These results suggested that TDF treatment could enhance the immunosuppressive effect of EVs.

**Fig 4:**
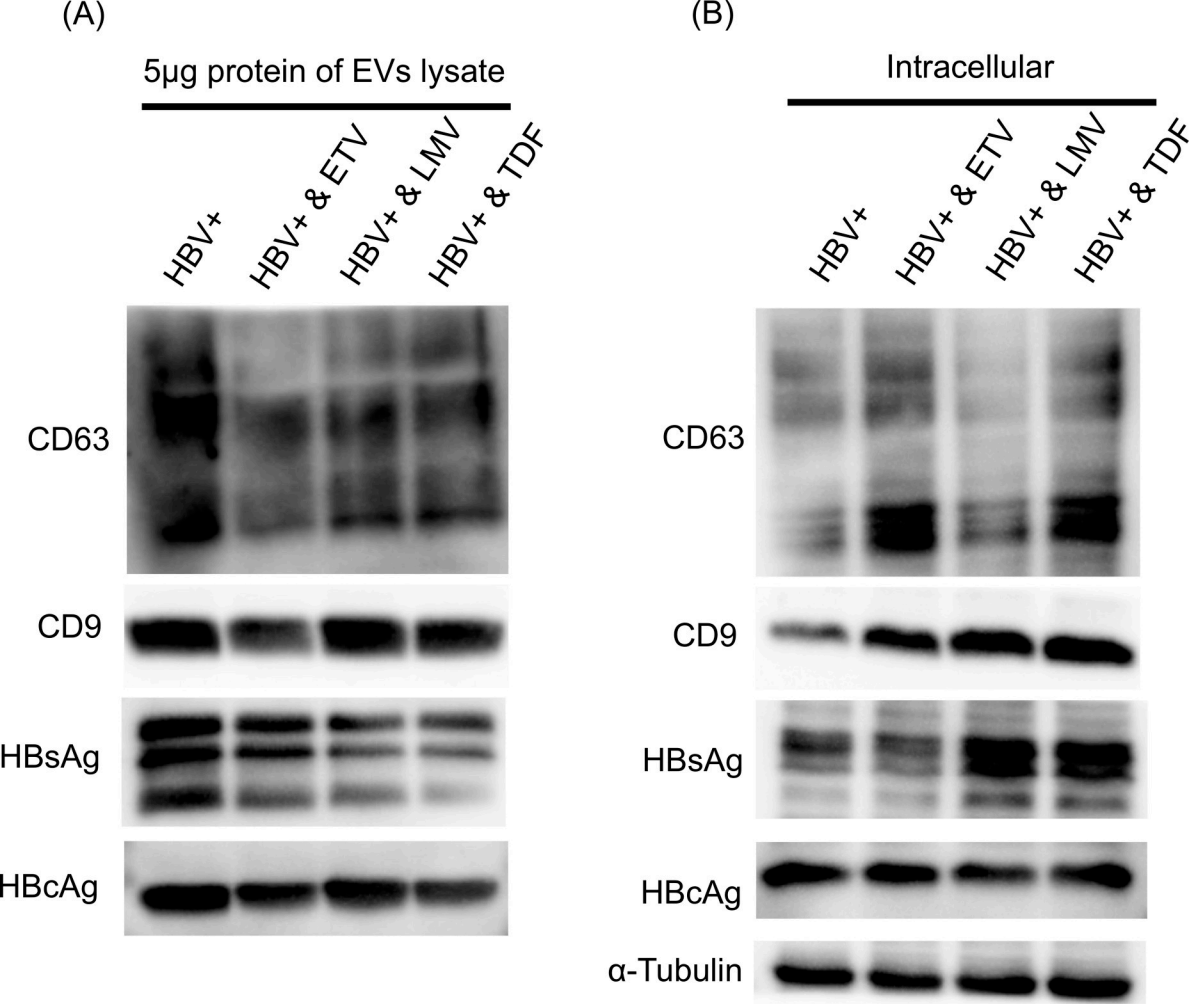
Abundance ratio of exosome markers and viral proteins in extracellular vesicles (EVs) and intracellular space after treatment with NRTIs. (A) HepAD38 cells with hepatitis B virus (HBV) replication were treated with entecarvir (ETV), lamivudine (LMV), and tenofovir (TDF) for 9 days. Then, EVs were collected by ultracentrifugation from 10 mL of culture supernatant of HepAD38 cells treated with each NRTIs. Collected EVs were lysed in radioimmunoprecipitation assay buffer, and protein concentration was measured using DC protein assay. Abundance ratio of CD9, CD63, HBsAg, and HBcAg in 5 mg protein from each EV lysate were examined by western blotting (representative of two experiments). (B) Abundance ratio of intracellular CD9, CD63, HBsAg, and HBcAg expression after NRTIs treatments were examined by western blotting (representative of two experiments), and α-tubulin was used as a loading control.

Next, we hypothesised that this enhancement of immunosuppressive effect was induced by changes in the profiles of the exosomes, subviral particles, and virions. Therefore, we investigated the profiles of exosome markers and viral proteins after the NRTIs treatments. Fig 4B–4D shows the representative chemiluminograms for each protein. Our initial analysis revealed the presence of CD9 and CD63 in F2 and F3 of the cells treated with ETV (Fig 3B). A similar profile was observed for cells treated with LMV and TDF (Fig 3C and 3D). We then probed for the presence of HBsAg and HBcAg in these samples. HBsAg was detected mainly in F4–F10 (Fig 3B–3D). HBcAg was detected mainly in F8–F10 (Fig 3B–3D). Altogether, the profiles of the exosome markers and viral proteins after the NRTIs treatments were similar to the profiles of the cells before the NRTIs treatments (Fig 2A).

### NRTIs treatments changed the composition of EVs secreted from HepAD38 cells with HBV replication and increased pgRNA secretion

Next, to further examine the composition of EVs after the NRTIs treatments, we investigated the abundance ratio of exosome markers and viral proteins in EVs and intracellular space after the NRTIs treatment. In EVs, the abundance ratio of exosome markers and viral proteins were reduced after NRTIs treatment (Fig 4A). On the contrary, in the intracellular space, the abundance ratio of exosome markers and HBsAg increased after the NRTIs treatments (Fig 4B). These results suggested that the NRTIs treatment inhibited secretion of exosomes, subviral particles, and virions.

**Fig 5:**
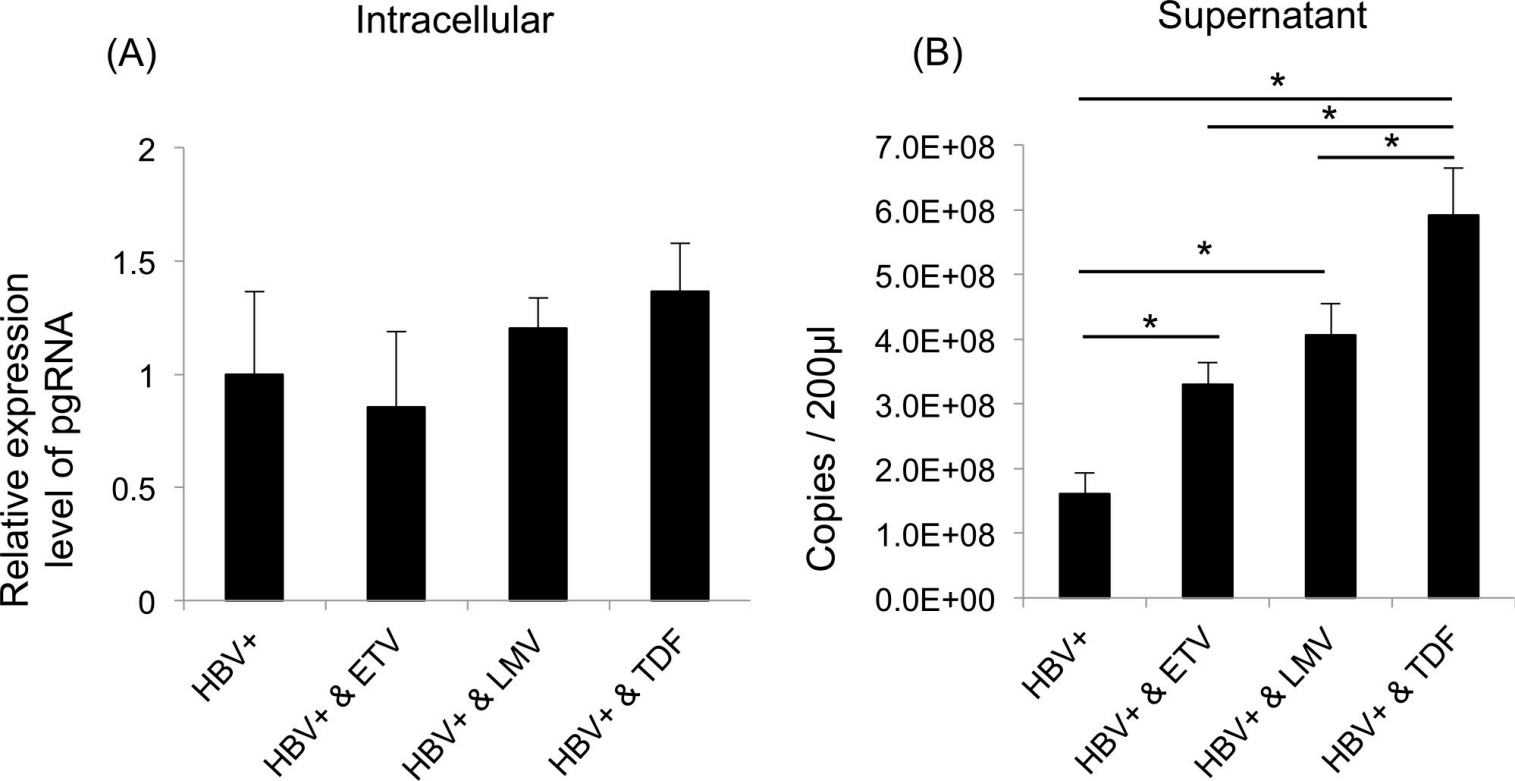
The level of pgRNA in the supernatant and intracellular space of HepAD38 cells with hepatitis B virus (HBV) replication after NRTIs treatment. (A) Total RNA from HepAD38 cells with HBV replication after the NRTIs treatments was collected. Then, the level of pgRNA was measured by quantitative RT-PCR. The expression was normalised to that of GAPDH. (B) Viral RNA from the supernatant of HepAD38 cells with HBV replication after the NRTIs treatments was collected. Then, the level of pgRNA was measured by quantitative RT-PCR. All values are presented as the mean ± S.E.M. of three biological replicates (n = 3) (*p < 0,05).

NRTIs can significantly inhibit HBV replication and decrease serum HBV DNA to undetectable level. However, HBsAg and HBcAg were detected in the EVs after the NRTIs treatments (Figs 3B and 4A). Previously, it has been reported that pregenomic RNA (pgRNA) virion levels increased in the serum of chronic hepatitis B patients after NRTIs treatments [7]. Therefore, we examined pgRNA levels in the supernatant and intracellular space of HepAD38 cells with HBV replication after the NRTIs treatments. HepAD38 cells with HBV replication were treated with ETV, LMV, and TDF for 9 days. Then, the level of pgRNA in the supernatant and intracellular space were investigated by qPCR. The intracellular level of pgRNA remained unchanged after the ETV, LMV, and TDF treatments (Fig 5A). On the contrary, the level of pgRNA in the supernatant increased when HepAD38 cells were treated with ETV, LMV, and TDF (Fig 5B). In particular, the level of pgRNA was the highest when HepAD38 cells were treated with TDF (Fig 5B). These results were consistent with a previous study, suggesting that enhancement of immunosuppressive effect after TDF treatment is caused by changes in EV composition or an increase of pgRNA in the supernatant.

## Discussion

HBV infected hepatocytes secreted various kinds of the EVs such as HBV virion, HBs particles, psuedovirion, and exosomes. Here, we showed that these particles upregulates PD-L1 in the monocytes. PD-L1 is immunomodularoty molecules which induce suppression of T cells expressing PD-1. Its blockade by the antibody revolutionized the cancer immune therapy. As the expression of PD-L1 on monocytes, antigen presenting cells (APCs), also significantly affects these functions, the EVs critically affect host immune system [24]. Each fractionation containing HBs particles, exosome, and virion upregulated PD-L1. ETV, TDF, and LAV affected the composition of the EVs, resulting that the EVs collected from the TDF treated HepAD38 farther upregulated PD-L1.

The exosomes secreted from HBV infected hepatocytes have been reported to be involved in antiviral innate immune response against HBV. The exosome induces NKG2D ligand expression in macrophages by stimulating MyD88, TICAM-1, and MAVS-dependent pathways, suggesting the importance of exosomes for NK cell activation. [18]. In contrast, infection of hepatocytes with HBV increased immunoregulatory microRNA levels in EVs and exosomes, which were transferred to macrophages, thereby suppressing IL-12p35 mRNA expression in macrophages to counteract the host innate immune response [25]. The target cells which take up the EVs secreted by HBV infected hepatocytes are macrophages which is compatible with our results. While their roles of the exosome generally antiviral innate immune response agaist HBV, ours seems mainly immunoregulatory. The difference might come from the sourse of macrophages: ours are the monocyte in human PBMCs, while theirs are mouse Kupper cells in the liver. Alternatively the cells which the exosomes were collected from are different; the cells we used for collection are mainly HepAD38 cells which express HBV by tet inducible promoter, while those they used mainly HepG2 cell infected by pHBV. driven by HBV endogenous promoter.

APCs-expressed PD-L1 ligation with its receptor PD-1 on T cells has been reported to stimulate interleukin-10 production [26]. In the recent studies of HBV and HCV- infected patients, correlation was shown between PD-L1 expression on monocytes and liver inflammation. PD-L1/CD86 ratio in the CD14^++^CD16^+^ monocyte positively correlated with the HCV load and core antigen in the chronic HCV infection [27]. Moreover, upregulation of PD-L1 on Kupffer cells and infiltrating monocytes/macrophages was closely associated with liver inflammation and antiviral immunity [28, 29]. Based on these studies, upregulated PD-L1 on monocytes after EVs uptake might be involved in the progression of HBV infection possibly by suppression of T cells.

Though CD9 and CD63 which are marker of exosomes are accumulated in the cell, those in the exosome decreased in the treatment of NRITs. Similarly while the protein expression of HBc was comparable between the cells treated with and without NRITs, that of the exosomes and HBs particles recduced. These results suggest that the biogenesis of these particles might be affected by NRITs. Among the NRITs, EVs collected from the TDF treated HepAD38 were most potent in upregulation of PD-L1 on monocytes (Fig 3A). pgRNA levels in the culture supernatant was highest after treatment with TDF treatment. (Fig 5). The correlation of the results should be further investigated.

During treatment with NRITs, HBsAg is continuously produced from HBV infected cells. Therefore, it is presumed that HBV-infected hepatocytes is identified and eliminated by the host immune system. However, in the clinical settings, the amount of HBsAg in the serum sometimes remains unchanged in the patients treated with NRITs, though HBV DNA is undetectable, suggesting that few cccDNA positive hepatocytes are eliminated. [30, 31]. The upregulation PD-L1 on monocytes taking up EVs secreted from hepatocytes might be responsible for the clinical observation.

The combination therapy for HBV of NRITs and a blockade of the PD-1 and PD-L1 interaction by an antibody showed promising results [32]. Considering the results of our study, this combination therapy has the potential to cause a dramatic reduction of relapse and fulminant hepatitis, after discontinuation of ETV. Further study will be needed.
